# The Association Between the Uncoupling Protein-1 Gene A-3826G Polymorphism and High-density Lipoprotein Cholesterol in A General Japanese Population: A Consideration of the Obesity Status

**DOI:** 10.4021/jocmr738w

**Published:** 2011-11-10

**Authors:** Kazuhiko Kotani, Shinji Fujiwara, Kokoro Tsuzaki, Yoshiko Sano, Narumi Nagai, Toshiyuki Yamada, Naoki Sakane

**Affiliations:** a Division of Preventive Medicine, Clinical Research Institute, National Hospital Organization Kyoto Medical Center, Kyoto, Japan; b Department of Clinical Laboratory Medicine, Jichi Medical University, Shimotsuke-Tochigi, Japan; c Department of Food Science and Nutrition, University of Hyogo, Hyogo, Japan

## Abstract

**Background:**

Limited studies have shown inconsistent data about the association between the uncoupling protein 1 (UCP1) gene A-3826G polymorphism and high-density lipoprotein (HDL) cholesterol levels. The present study investigated the association between the A-3826G polymorphism and low HDL-cholesterolemia in non-obese and obese subjects.

**Methods:**

Anthropometric and biochemical factors, in addition to genotyping by an allele-specific DNA assay, were measured in 294 community-dwelling Japanese subjects (male/female: 127/167, mean age: 65 years). Obesity was defined as a body mass index (BMI) ≥ 25 kg/m^2^, and low HDL-cholesterolemia was defined as < 1.04 mmol/L of HDL-cholesterol.

**Results:**

The subjects with the G/G genotype (n = 27) showed a significantly higher prevalence of low HDL-cholesterolemia (37%) than those with the A/A + A/G genotype (13%) in the obese group (n = 102). There was a non-significant difference in the prevalence of low HDL-cholesterolemia between subjects with the G/G genotype (n = 45, 13%) and with the A/A + A/G genotype (15%) in the non-obese group (n = 192). A multivariate-adjusted logistic regression analysis of the presence of low HDL-cholesterolemia revealed that carrying the G/G genotype was an independent and significant factor positively associated with low HDL-cholesterolemia [odds ratio (OR): 6.85, 95% confidence interval (CI): 1.65-28.49] in the obese group, while carrying the G/G genotype exhibited a non-significant but reduced OR, by one-half, for low HDL-cholesterolemia (OR: 0.51, 95% CI: 0.13-1.96) in the non-obese group.

**Conclusions:**

The obesity status could have opposing impacts on the relationship between the G/G genotype and low HDL-cholesterolemia, providing insight into the need to consider the obesity levels when studying the association between the UCP-1 gene A-3826G polymorphism and HDL-cholesterol.

**Keywords:**

Obesity; Body mass index; HDL-C; Atherosclerotic risk

## Introduction

Obesity is a global health problem due to its epidemic proportions and the detrimental consequences associated with obesity [1,2]. Uncoupling protein 1 (UCP1) is a mitochondrial component and a proton transporter, which uncouples oxidative metabolism from ATP synthesis, and dissipates energy through heat production [3,4]. UCP1 plays important roles in energy homeostasis, and UCP1 gene polymorphisms have been implicated in the pathogenesis of obesity and related metabolic disorders, including lipid disorders [3,4]. An A-3826G polymorphism within the promoter region of the UCP-1 gene is a candidate gene polymorphic site related to these disorders [5].

The blood levels of high-density lipoprotein (HDL) cholesterol (HDL-C) are a major atherosclerotic risk marker [6,7]. The HDL-C concentrations are generally determined by the interplay of environmental and genetic factors [7]. Few human studies have directly focused on the relationship between the UCP-1 gene A-3826G polymorphism and HDL-C levels, but inconsistent data on this relationship have been reported in several cross-sectional association studies [8-15]. Although carrying the G allele or G/G genotype was reportedly associated with lower HDL-C levels [9,11,13], we found no association between the A-3826G polymorphism and HDL-C levels [8,10,12,14] as well as a possibly protective association of the G/G genotype with low HDL-cholesterolemia [15]. The UCP-1 expression is regulated by the condition of the individual, including the extent of obesity, and carrying the G allele is related to a reduced UCP-1 expression in obese subjects [9]. Therefore, the inconsistencies reported between studies on the association between the A-3826G polymorphism and HDL-C levels [8-15] may be, at least in part, explained by the difference in the studied populations, such as differences in the composition of obese and non-obese subjects. In fact, we noted that all of the studies showing that carrying the G allele or G/G genotype was significantly associated with lower HDL-C levels were in obese populations {i.e., 42-47 kg/m^2^ [9], 33 kg/m^2^ [11], and 34 kg/m^2^ [13] for the mean body mass index (BMI)} except one study (mean BMI 45 kg/m^2^) [8], while studies showing no association between the A-3826G polymorphism and HDL-C [10,12,14] and carrying the G/G genotype as a protective factor for low HDL-cholesterolemia [15] were for relatively non-obese populations {i.e., mean BMI 25.5 kg/m^2^ (in Caucasians) [10], 22 kg/m^2^ [12], 22 kg/m^2^ [14], and 22-23 kg/m^2^ [15]}. Based on this background information, the present study aimed to compare the association between the UCP-1 gene A-3826G polymorphism and low HDL-cholesterolemia in a general Japanese population by analyzing subject-groups separated according to their obesity status.

## Methods

This cross-sectional association study included a total of 294 community-dwelling Japanese subjects [127 males and 167 females, mean age 65 ± 13 (SD) years], recruited from community-based health check-ups conducted in the Mima area of Japan. The study was approved by the institutional ethics committee and each subject gave informed consent. The subjects who were basically asymptomatic and not taking any medication were eligible. Excluded were subjects who suffered from any acute inflammatory disease, or had a clinical history of cardiovascular, thyroid, malignant, severe hepatic or renal diseases.

Smoking habits were determined as a current or non-smoker through a self-reported questionnaire. In addition to the BMI, calculated as the body weight (kg) divided by the square of the height (m), the systolic blood pressure (SBP) and diastolic blood pressure (DBP) were measured in the seated subject’s right arm using a mercury sphygmomanometer. Blood was sampled after an overnight fast. The serum total cholesterol (T-chol), triglycerides (TG) and HDL-C levels were enzymatically measured. Hemoglobin A1c (HbA1c) levels were measured using a high performance liquid chromatography method. Genomic DNA was extracted from the subject’s buccal mucosa cells obtained using cytobrushes, and genotypes were determined by an intercalater-mediated fluorescent allele-specific PCR method using specific primers, as described previously [8].

The data were presented as the means ± standard deviation (SD) or the medians plus interquartile range. The genotype and allele frequencies for Hardy-Weinberg equilibrium were examined using the χ^2^-test. Differences between the genotype-based groups were compared using either the unpaired t-test, one way ANOVA (with a multi-comparison test), or χ^2^-test (with a residual analysis) in each group of non-obese and obese subjects. When the two groups by genotype were compared, we divided subjects into the A/A + A/G genotype group and the G/G genotype group according to the earlier study [15]. The TG values were log-transformed because of their skewed distribution in this analysis. A multivariate logistic regression analysis on the presence of low HDL-cholesterolemia, adjusted for all the measured factors including the G/G genotype (reference: the A/A + A/G genotype), was performed to observe whether the genotype was associated with low HDL-cholesterolemia in each group of non-obese and obese subjects. Obesity was defined as a BMI ≥ 25 kg/m^2^ according to the clinical guideline of the Japan society for the study of obesity [16], and low HDL-cholesterolemia was defined as HDL-C < 1.04 mmol/L according to the clinical guideline of the Japan Atherosclerosis Society [17]. A P-value < 0.05 was considered to be significant.

## Results

Overall, the distributed number of A/A, A/G and G/G genotypes was 91 (31%), 131 (45%) and 72 (24%), respectively, among all subjects (n = 294). The frequency of the G-allele was 47%. This frequency was similar to earlier Japanese reports [12,14,15]. These frequencies were in Hardy-Weinberg equilibrium (χ^2^ = 1.64, P = 0.45). In the non-obese group (n = 192), the subject number of A/A, A/G and G/G genotypes was 59 (31%), 88 (46%) and 45 (23%), while in the obese group (n = 102), the subject number of A/A, A/G and G/G genotypes was 32 (31%), 43 (42%) and 27 (27%), respectively. There was no significant difference in the genotype distribution between the non-obese and obese groups (P = 0.79).

When the clinical characteristics of subjects were compared between the respective genotype carriers, significant differences in HDL-C levels (A/A genotype: 1.37 ± 0.41 mmol/L, A/G: 1.42 ± 0.31 mmol/L, G/G: 1.20 ± 0.30 mmol/L; F = 3.54, P = 0.03) and the prevalence of low HDL-cholesterolemia (A/A genotype: 5 subjects, 16%, A/G: 4 subjects, 9%, G/G: 10 subjects, 37%; F = 0.461, P = 0.01) were observed in the obese group. There were significant differences in HDL-C levels between the A/G genotype and G/G genotype carriers (P = 0.03) and the prevalence of low HDL-cholesterolemia between the A/G genotype and G/G genotype carriers (P = 0.01) in this group. The other measured factors did not show any significant differences between the respective genotype carriers in the non-obese and obese groups (data not shown).

The clinical characteristics of the two groups by genotype (the A/A + A/G genotype group versus the G/G genotype group) are listed in each group of non-obese and obese subjects in Table 1. In the obese group, the G/G genotype carriers had significantly lower HDL-C levels and a significantly higher prevalence of low HDL-cholesterolemia than the A/A + A/G genotype carriers. The other measured factors did not show any significant differences between the two groups by genotype in the non-obese and obese groups.

**Table 1:** The Clinical Characteristics of Subjects Based on the Genotypes of the Uncoupling Protein-1 Gene A-3826G Polymorphism in the Non-obese and Obese Groups

**Genotype**	**Non-obese**				**Obese**		
		
	**A/A + A/G**	**G/G**	**P value**	****	**A/A + A/G**	**G/G**	**P value**
Subject number (n)	147	45	-		75	27	-
Age (years)	66 ± 12	66 ± 13	0.98		63 ± 14	62 ± 15	0.72
Male (n)	67 (46%)	21 (47%)	0.90		30 (40%)	9 (33%)	0.55
Current smoker (n)	23 (16%)	8 (18%)	0.73		9 (12%)	3 (11%)	0.90
BMI (kg/m^2^)	22.2 ± 2.0	22.3 ± 1.9	0.64		27.1 ± 1.9	27.4 ± 2.3	0.48
SBP (mmHg)	139 ± 20	135 ± 19	0.26		139 ± 21	138 ± 17	0.92
DBP (mmHg)	77 ± 10	74 ± 10	0.08		78 ± 13	80 ± 13	0.52
T-chol (mmol/L)	4.85 ± 0.87	4.94 ± 1.13	0.69		4.86 ± 0.85	4.72 ± 0.97	0.48
HDL-C (mmol/L)	1.44 ± 0.38	1.43 ± 0.38	0.94		1.39 ± 0.36	1.20 ± 0.30	0.01*
LHDLC (n)	22 (15%)	6 (13%)	0.79		9 (13%)	10 (37%)	< 0.01*
TG (mmol/L)	0.98 (0.75-1.23)	0.98 (0.76-1.52)	0.24		1.05 (0.84-1.51)	1.15 (0.86-1.39)	0.86
HbA1c (%)	5.6 ± 1.3	5.6 ± 1.3	0.97		5.6 ± 0.7	5.5 ± 0.6	0.85

BMI: body mass index; SBP: systolic blood pressure; DBP: diastolic blood pressure; T-chol: total cholesterol;, HDL-C: high-density lipoprotein cholesterol; LHDLC: low high-density lipoprotein cholesterolemia; TG: triglycerides; HbA1c: hemoglobin A1c. The data are expressed as the means ( standard deviation, medians (interquartile range) or subject number (%). Differences in data between the genotype-based groups were examined by the unpaired t-test or χ^2^-test within each group of non-obese and obese subjects. The TG values were log-transformed because of their skewed distribution in the analysis. Significant level: *: P < 0.05.

Subsequently, the results of the multivariate-adjusted logistic regression analysis of the presence of low HDL-cholesterolemia are shown in Table 2. In the obese group, the analysis revealed that carrying the G/G genotype, as well as the BMI and TG, was an independent and significant positive factor associated with low HDL-cholesterolemia, while the SBP and T-chol showed an independent and significant inverse association with low HDL-cholesterolemia. In the non-obese group, the same analysis revealed that the TG showed an independent and significant positive association and the T-chol showed an independent and significant inverse association with low HDL-cholesterolemia, while carrying the G/G genotype exhibited a non-significant but reduced (by one-half) odds ratio (OR) for the association with the presence of low HDL-cholesterolemia.

**Table 2:** The Results of the Multivariate-adjusted Logistic Regression Analysis of Factors Associated With Low High-density Lipoprotein Cholesterolemia in the Non-obese and Obese Groups

**Factor**	**Non-obese**		****	**Obese**	
		
	**OR (95% CI)**	**P value**	****	**OR (95% CI)**	**P value**
Age (years)	1.04 (0.98-1.09)	0.19		1.04 (0.98-1.11)	0.22
Gender (male)	3.05 (0.86-10.78)	0.08		0.95 (0.19-4.75)	0.95
Current smoking (presence)	1.72 (0.43-6.83)	0.44		6.51 (0.71-59.9)	0.1
BMI (kg/m^2^)	0.94 (0.70-1.27)	0.69		1.53 (1.05-2.21)	0.03*
SBP (mmHg)	0.98 (0.94-1.02)	0.32		0.94 (0.89-0.99)	0.03*
DBP (mmHg)	1.02 (0.94-1.10)	0.63		1.06 (0.98-1.16)	0.15
T-chol (mmol/L)	0.95 (0.93-0.97)	< 0.01*		0.95 (0.92-0.99)	< 0.01*
TG (mmol/L)	1.03 (1.02-1.05)	< 0.01*		1.02 (1.01-1.03)	< 0.01*
HbA1c (%)	1.14 (0.79-1.65)	0.49		1.67 (0.51-5.46)	0.4
G/G genotype (presence)	0.51 (0.13-1.96)	0.33		6.85 (1.65-28.49)	< 0.01*

OR: odds ratio; CI: confidence interval; BMI: body mass index; SBP: systolic blood pressure; DBP: diastolic blood pressure; T-chol: total cholesterol; HDL-C: high-density lipoprotein cholesterol; TG: triglycerides; HbA1c: hemoglobin A1c. All the listed factors, including the G/G genotype of the uncoupling protein-1 gene A-3826G polymorphism (reference: the A/A + A/G genotypes), were entered into the multivariate-adjusted logistic regression analysis models. Significant level: *: P < 0.05.

## Discussion

The present study showed that the G/G genotype of the UCP-1 gene A-3826G polymorphism was an independent and significant factor positively associated with low HDL-cholesterolemia in the obese group, while the G/G genotype was a relatively protective factor associated with low HDL-cholesterolemia in the non-obese group. Even though the results of the OR for the G/G genotype on low HDL-cholesterolemia in the non-obese group did not reach a statistically significant level, the impact of the reduction of the OR (by one-half) with regard to low HDL-cholesterolemia should not be ignored. The results of the obese group are similar with the previous studies [9,11,13], and the results of the non-obese group likely confirm the earlier study [15]. Blood levels of HDL-C are a major atherosclerotic risk marker, and the association between HDL-C metabolism and genetic factors remains to be explored. It is therefore important to note that there may be an opposite trend for the association between the UCP-1 gene A-3826G polymorphism and low HDL-cholesterolemia based on the obesity status of subjects. This may provide a possible explanation for the inconsistent data shown for this association in the previous studies [8-15] and it may also provide further understanding of the different roles of the UCP-1 gene A-3826G polymorphism under obese and non-obese conditions.

The precise mechanisms underlying the present findings are unclear. UCP-1 is a mitochondrial protein, potentially associated with energy homeostasis [3,4]. UCP-1 is basically expressed in brown adipose tissue, and recent studies have shown that adult humans possess metabolically active brown adipose tissue [18-20]. Changes in the oxidation of free fatty acids in the mitochondria can alter the blood levels of lipids through tissue cholesterol transport; thus, this pathway to control blood lipids may be affected by genetic polymorphisms such as the UCP-1 gene A-3826G polymorphism [13]. The UCP-1 expression is regulated by the extent of obesity, and the G allele has been reported to be related to a reduction of the expression of the gene in an obese population [9]. This may be one reason for the present finding that the G/G genotype is positively associated with low HDL-cholesterolemia in obese subjects. On the other hand, the UCP-1 expression remains to be incompletely examined in non-obese populations including lean subjects [9]. There may be a difference in the UCP-1 expression and the role of the A-3826G polymorphism in regulating the levels of blood lipids between obese and non-obese subject-populations. Such a difference may partially account for the present study findings.

In the present study, the TG (in the non-obese and obese groups) and BMI (in the obese group) were factors that were positively associated with low HDL-cholesterolemia. These associations have been well-recognized [21]. In addition, that the T-chol (in the non-obese and obese groups) was a factor inversely associated with low HDL-cholesterolemia is plausible, because HDL-C participates in regulating the T-chol levels. Although the SBP (in the obese group) was a factor that was inversely associated with low HDL-cholesterolemia, this appeared to be paradoxical, because low HDL-cholesterolemia is an atherosclerotic risk factor. The reason for this relationship was not determined, so further studies, including the SBP-related confounders, will be required.

The present study had a few limitations. The sample size was relatively small, and there was not a very high prevalence of low HDL-cholesterolemia. This might have lead to the non-significance of the association between the G/G genotype and low-HDL-cholesterolemia in the non-obese group. The cross-sectional design of the study did not fully allow for determination of the cause-and-effect relationship between factors. Moreover, although there have been some reports showing the association between HDL-C levels and genetic variations of the other types of uncoupling proteins such as UCP-2 and UCP-3 [22,23], no data on such types of uncoupling proteins were available in this study. Additional studies with larger populations, prospective designs and various genetic polymorphisms are needed.

In summary, carrying the G/G genotype of the UCP-1 gene A-3826G polymorphism was an independent and significant factor positively associated with low HDL-cholesterolemia in obese subjects, and in contrast, carrying the G/G genotype was a relatively protective factor associated with low HDL-cholesterolemia in non-obese subjects. The present findings may provide an explanation for the inconsistent data obtained in the previous studies (of populations with a different status of obesity) regarding the association between the UCP-1 gene A-3826G polymorphism and HDL-C, and may provide a hint about the different roles of the UCP-1 gene A-3826G polymorphism under obese and non-obese conditions. Further studies are warranted to confirm these results and to clarify the biological mechanism(s) responsible for the observed association.
